# Standardized *Apis mellifera* Propolis Extract (EPP-AF^®^) Reduces Egg Burden and Granulomatous Inflammation in Experimental *Schistosoma mansoni* Infection

**DOI:** 10.3390/pathogens15090980

**Published:** 2026-09-15

**Authors:** Vitor Augusto Ferreira dos Santos, Irlla Correia Lima Licá, Gleycka Cristine Carvalho Gomes Frazão, Ranielly Araujo Nogueira, Maria Gabriela Sampaio Lira, João Gustavo Mendes Rodrigues, Guilherme Silva Miranda, Josivan Regis Farias, Sulayne Janayna Araújo Guimarães, Mayara Cristina Pinto da Silva, Thiare Silva Fortes, Lucilene Amorim Silva, Wallyson André dos Santos Bezerra, Alexandra Martins dos Santos Soares, Rosane Nassar Meireles Guerra, Flávia Raquel Fernandes Nascimento

**Affiliations:** 1 Laboratory of Immunophysiology, Center for Biological and Health Sciences, Federal University of Maranhão, Saint Louis 65080-805, MA, Brazil; 2 Laboratory of Biology, Department of Education, Federal Institute of Education, Saint Raymond of Mangabeiras 65840-000, MA, Brazil; 3 Laboratory of Pathology and Immunoparasitology, Center for Biological and Health Sciences, Federal University of Maranhão, Saint Louis 65080-805, MA, Brazil; 4 Laboratory of Applied Cancer Immunology, Center for Biological and Health Sciences, Federal University of Maranhão, Saint Louis 65080-805, MA, Brazil

**Keywords:** schistosomiasis, *Schistosoma mansoni*, cytokines, EPP-AF^®^, inflammation, granuloma

## Abstract

Propolis has potential anthelmintic activity against *Schistosoma mansoni*, although its mechanisms remain poorly characterized. This study evaluated the biological effects of standardized *Apis mellifera* propolis extract (EPP-AF^®^) in experimental murine schistosomiasis. In vitro, EPP-AF^®^ reduced adult worm viability and motility. In vivo, Swiss mice were allocated to four groups: EPP-AF^®^ (4.5 mg/kg/day, orally, for 30 days), Praziquantel (PZQ; 50 mg/kg, days 45–51 post-infection), infected untreated control, and uninfected untreated SHAM control. EPP-AF^®^ delayed oviposition and reduced egg production and tissue retention, granuloma formation, hepatic fibrosis, and eosinophilia. It also modulated splenic cytokine production, reducing inflammatory mediators and increasing IL-10. Molecular docking indicated favorable interactions between propolis phenolic compounds and SmCB1. Collectively, EPP-AF^®^ exhibited antiparasitic and tissue-protective effects during experimental *S. mansoni* infection, supporting its pharmacological potential against schistosomiasis.

## 1. Introduction

Schistosomiasis is a parasitic disease considered one of the most significant neglected tropical diseases (NTDs), with high prevalence in Africa, Asia, and Latin America. In 2024, the World Health Organization (WHO) reported its occurrence in 79 countries [1,2]. Global data from 2024 indicate that at least 253.7 million people required preventive interventions for schistosomiasis control [2,3].

In the vertebrate host, early infection may present with dermatological and systemic symptoms such as fever, abdominal pain, and headache [4,5]. The disease is caused by trematode worms of the genus *Schistosoma*, with *S. mansoni* being the only species reported in Brazil to date. Before egg deposition, a Th1-polarized immune response predominates, characterized by increased production of pro-inflammatory cytokines such as IFN-γ, IL-2, IL-1, IL-12, IL-6, and TNF-α [6,7]. However, during the acute phase, parasite eggs become trapped in tissues, triggering granulomatous inflammation and progressive fibrosis, which may compromise organ function. In the chronic phase, a Th2-dominated immune response prevails, with elevated IL-4, IL-5, IL-10, and IL-13 levels. This shift promotes eosinophilia, M2 macrophage activation, IgE production, and enhanced egg migration and retention in host tissues. As a result, tissue architecture and function—particularly in the liver—are often severely compromised, leading to hepatomegaly and fibrosis [2,5,7].

Currently, PZQ remains the only WHO-recommended treatment for schistosomiasis, nearly four decades after its introduction [8]. However, recent studies report several limitations, including side effects, reduced efficacy against juvenile worms, lack of protection against reinfection, and inability to reverse established fibrosis [9,10,11,12,13]. Moreover, repeated use in endemic regions raises concerns about the potential emergence of resistance [14].

Considering these limitations, natural products have gained increasing attention as sources of bioactive compounds for drug development and as potential adjuvants to improve existing therapies. Propolis, a resinous product collected by *Apis mellifera*, contains a wide variety of biologically active compounds with pharmacological activities, including antioxidant, anti-inflammatory, immunomodulatory, and antiparasitic effects [15,16]. Several studies have reported its use as a dietary supplement and immunological adjuvant [17,18,19], as well as its antiparasitic properties [20,21,22,23,24,25,26].

Among these properties, the anti-helminthic activity of propolis against *S. mansoni* has shown promising results [21,27,28]. However, despite evidence of its efficacy, the immunological effect responses and granuloma characteristics during the infection remain unclear. Therefore, the present study aimed to evaluate the effects of a standardized green propolis extract (*Apis mellifera*, EPP-AF^®^) on both the early and chronic stages of *S. mansoni* infection in mice. Particular attention was given to the effects of propolis on immune response, egg deposition, and the number and size of granulomas in target tissues.

## 2. Materials and Methods

### 2.1. Standardized Propolis Extract (EPP-AF^®^)

The standardized *A. mellifera* green propolis extract (EPP-AF^®^) used in this study was kindly provided by Apis Flora^®^ Ltd. (Ribeirão Preto, SP, Brazil), through a partnership with the Immunophysiology Laboratory at the Federal University of Maranhão. EPP-AF^®^ is a patented, commercially standardized product (Patent No. 0405.483-0), consisting of green propolis, neutral alcohol, and purified water, with a minimum concentration of 16% *w*/*v* dry extract (Batch No. 251400119LP). The commercial formulation contains 58.28% ethanol (*v*/*v*). High-performance liquid chromatography (HPLC) analysis identified caffeic acid (0.288–0.336 mg/g), p-coumaric acid (1.486–1.561 mg/g), trans-cinnamic acid (0.091–0.167 mg/g), aromadendrin (0.993–1.125 mg/g), isosakuranetin (2.256–2.265 mg/g), and artepillin C (6.962–8.533 mg/g) as the main chemical markers of EPP-AF^®^ [29]. Before administration, EPP-AF^®^ was diluted in water. The administered dose of 4.5 mg/kg was calculated based on the dry extract content of EPP-AF^®^, corresponding to the minimum concentration of 16% *w*/*v* in the commercial formulation. The ethanol content of the commercial formulation was not included in the administered dose calculation, as the dose was expressed based on the dry extract content of EPP-AF^®^.

### 2.2. In Vitro Assay: Direct Effect of Propolis on Adult Worms

Adult *S. mansoni* worms were recovered by portal perfusion using sterile saline solution (0.9% NaCl) from mice at 60 days post-infection [30]. The worms were suspended in RPMI-1640 medium supplemented with 100 μg/mL penicillin, 100 μg/mL streptomycin, and 10% fetal bovine serum. Worms were selected based on preserved morphology and spontaneous motility; those showing evident morphological alterations or complete immobility were excluded. Two adult worms, one male and one female, were placed in each well of a 24-well plate containing 2 mL of culture medium.

After 2 h of incubation at 37 °C and 5% CO_2_, EPP-AF^®^ was added at final concentrations of 50, 150, and 250 μg/mL, selected based on a preliminary pilot assay. Culture medium alone was used as the negative control, and PZQ (10 μg/mL) as the positive control. A separate vehicle control was not included.

Worm viability was assessed every 6 h for up to 60 h based on spontaneous motility and morphological integrity [31,32]. Worms exhibiting active and coordinated movement with preserved morphology were considered viable, whereas those showing complete immobility and a straight body posture were considered non-viable. Each experiment was performed in four independent replicates, with samples analyzed in triplicate [32].

### 2.3. In Vivo Assay: Infection and Treatment of Mice with EPP-AF^®^

Male Swiss mice (*n* = 24), aged 2–3 months and weighing approximately 35 g, were obtained from the Central Animal Facility of the Federal University of Maranhão (São Luís, Brazil). All procedures were approved by the Animal Ethics Committee of the Federal University of Maranhão on 21 February 2020 (CEUA/UFMA, Protocol No. 23115.002254/2020-18). The animals were randomly assigned to four groups (*n* = 6 per group): Sham, consisting of uninfected and untreated animals; Control, consisting of infected and untreated animals; PZQ, consisting of infected animals treated orally with PZQ (50 mg/kg) from days 45 to 51 post-infection [33]; and EPP-AF^®^, consisting of infected animals treated orally with EPP-AF^®^ (4.5 mg/kg/day) for 30 consecutive days, beginning immediately after infection.

Infection was induced by subcutaneous inoculation of 70 viable *S. mansoni* cercariae per animal [34]. The LE strain of *S. mansoni* was used in the present study. EPP-AF^®^ was administered orally once daily from days 0 to 30 post-infection. The dose of 4.5 mg/kg/day was selected based on previous pilot studies and calculated according to the dry extract content. Before administration, EPP-AF^®^ was dissolved in water and administered at a volume of 200 μL per animal. Animals were evaluated at 30 and 90 days post-infection. Peripheral blood samples were collected on days 30 and 90 for differential leukocyte counts, and fecal samples were collected from days 30 to 90 to monitor egg shedding. On day 90 post-infection, animals were euthanized by intramuscular administration of ketamine:xylazine:PBS (2:1:1) solution, and whole blood, peritoneal cells, bone marrow, spleen, liver, intestine, and kidneys were collected. Histopathological and histomorphometric analyses, splenic cellularity assessment, and flow cytometry were performed, and splenic cells were obtained for cytokine analysis. All experiments were performed independently and repeated twice to confirm the reproducibility of the results.

#### 2.3.1. Egg Quantification in Feces, Liver, and Intestine

On day 30 post-infection, fecal samples were collected to monitor *S. mansoni* egg shedding during the infection, and leukocyte counts were performed. Fecal samples were weighed and homogenized in 10 mL of 10% buffered formalin. Two 100 μL aliquots of the fecal suspension were examined by optical microscopy, and the number of *S. mansoni* eggs was recorded and expressed as eggs per gram of feces [35]. On day 90 post-infection, the animals were euthanized, and whole blood, peritoneal lavage fluid, bone marrow cells, splenocytes, and organs (liver, intestine, and kidneys) were collected for subsequent analyses.

For tissue egg quantification, the entire largest liver lobe from each animal was removed during necropsy and processed individually. The liver tissue was digested in 5% potassium hydroxide (KOH) according to the method described by [36], and the digested material was examined by optical microscopy for *S. mansoni* egg counting. The same procedure was applied to intestinal tissue, using 15 cm segments of the small and large intestines, which were processed separately for egg quantification.

#### 2.3.2. Histopathological Analysis of Liver, Intestine, and Kidney

Tissue samples from the liver (central portion of the bilobed right lobe), intestine (segments of the small and large intestine), and right kidney were fixed in 10% formalin for 48 h. Samples were subsequently washed, stored in 70% ethanol, dehydrated in graded alcohols, cleared in xylene, and embedded in paraffin. Histological sections were stained with hematoxylin and eosin (HE) [37] and examined by light microscopy using a Axio Scope.A1® (Carl Zeiss, Jena, Germany) photomicroscope. Histopathological parameters included egg presence and extent, granuloma presence and extent, fibrosis, tissue damage, necrosis, inflammatory infiltrate, and vascular congestion. Each parameter was scored using an ordinal scale adapted from Libério et al. [38]: absent (0), mild (1), moderate (2), or severe (3). The scores reflected the intensity or extent of each histopathological alteration, with higher scores indicating greater intensity or extent of the observed alteration. The same scoring criteria were consistently applied to all experimental groups.

#### 2.3.3. Granuloma Analysis in Liver, Intestine, and Kidney

Granulomas were additionally evaluated by histomorphometric analysis. Granuloma number and diameter were assessed in images obtained from six randomly selected fields per histological sample using a Zeiss Axio Scope.A1 photomicroscope. Images were analyzed using ImageJ software, version 6.0 (U.S. National Institutes of Health, Bethesda, MD, USA). Only isolated granulomas in the exudative phase containing a central miracidium were included in the morphometric analysis. The number of granulomas was expressed as the number of granulomas identified in the analyzed fields, whereas granuloma diameter was determined by morphometric analysis and expressed in mm.

#### 2.3.4. Leukocyte Count in Peripheral Blood

On day 30 post-infection, blood samples were collected through a tail incision, whereas on day 90 post-infection, blood samples were obtained via the retro-orbital route. On both occasions, 10 μL of blood were collected for analysis. Blood smears were prepared and stained using the Panótico Rápido kit (Laborclin, Brazil). The slides were examined by conventional light microscopy at 100× magnification, and differential leukocyte counts were performed by counting 100 cells per sample. The relative percentages of the different leukocyte populations were determined based on the total number of cells counted [39].

#### 2.3.5. Cellularity of Bone Marrow, Peritoneum, and Spleen

Peritoneal cells were obtained by intraperitoneal injection of 3 mL of sterile PBS. The cell suspension was processed using Cytospin, stained with a rapid panoptic kit (Laborclin, Brazil), and analyzed by light microscopy [40]. Bone marrow cells were collected by flushing the femur with 1 mL of PBS. Spleen cells were obtained by mechanical dissociation, filtered, and resuspended in 3 mL of PBS. For cell counting, bone marrow and spleen cell suspensions were mixed with crystal violet at a 9:1 ratio and transferred to a Neubauer hemocytometer. Cell counts were performed by light microscopy, and the results were expressed as cell concentration [41].

#### 2.3.6. Flow Cytometry Analysis of Splenic Cell Populations

Spleen cells (1 × 10^6^ cells/sample) were labeled with fluorochrome-conjugated monoclonal antibodies against CD3, CD19, F4/80, CD170/Siglec-F, and Ly6G. Anti-CD3 FITC (clone 145-2C11, Cat. No. 553062) was used to identify T cells, anti-CD19 FITC (clone 1D3, Cat. No. 557398) to identify B cells, anti-F4/80 FITC to evaluate macrophages, anti-CD170/Siglec-F FITC to evaluate the eosinophil-associated population, and anti-Ly6G PerCP-Cy5.5 (clone 1A8, Cat. No. 560602) to identify neutrophils. All antibodies were obtained from BD Biosciences Pharmingen (San Diego, CA, USA) and used according to the manufacturer’s instructions. A viability dye was used to discriminate viable from non-viable cells prior to flow cytometric analysis. Fluorochrome-matched isotype controls were included to assess nonspecific antibody binding and establish background fluorescence. A total of 10,000 events were acquired per sample using a Guava flow cytometer (EMD Millipore, Billerica, MA, USA) and the data were processed using FlowJo^®^ software, v.10.8.1 (BD Life Sciences, Ashland, OR, USA). The results were expressed as percentages of the respective splenic cell populations.

#### 2.3.7. Cytokine Quantification by Cytometric Bead Array (Cba)

Splenocytes (2 × 10^5^/well) were cultured in 96-well plates with complete RPMI-1640 medium (Sigma-Aldrich, St. Louis, MO, USA), supplemented with: 2 mM L-glutamine, 23 mM L-asparagine, 0.1 mM pyruvic acid, 1 mM folic acid, 1% streptomycin (100 μg/mL), 100 U/mL penicillin G, and 2% fetal bovine serum. Cultures were incubated for 48 h at 37 °C in 5% CO_2_ [42].

The levels of TNF-α, IFN-γ, IL-6, IL-2, IL-10, IL-4, IL-17, and MIP-1 were quantified in culture supernatants using the Th1/Th2/Th17 CBA kit (BD Biosciences Pharmingen, San Diego, CA, USA), per the manufacturer’s instructions. Samples were analyzed on a FACScalibur flow cytometer (BD Biosciences Pharmingen, San Diego, CA, USA). The results were processed with FlowJo^®^ software, v. 10.8.1 (BD Life Sciences, Ashland, OR, USA). Cytokine concentrations were expressed in pg/mL based on standard curves for each cytokine or chemokine.

### 2.4. Compound Structures and Molecular Docking

The structures of the compounds artepillin C, caffeic Acid, isosakuranetin, aromadendrin, p-coumaric acid, and cinnamic acid were obtained from the PubChem database (CIDs: 5472440, 689043, 160481, 122850, 637542, and 444539, respectively). All molecular structures were subsequently optimized geometrically in Avogadro [43] using the MMFF94 force field and the steepest descent minimization algorithm.

To investigate the predicted interactions of the compounds with the Cathepsin B1 protein from *S. mansoni* (SmCB1), molecular docking was carried out in the active site of SmCB1, using Molegro Virtual Docker 6.0 (MVD) software. The crystal structure of SmCB1 at 1.29 Å resolution (PDB ID: 6YI7, chain A), complexed with the inhibitor azanitrile, was retrieved from the Protein Data Bank (www.rcsb.org), and the cocrystallized inhibitor was used as a reference ligand.

Molecular docking was conducted using the MolDock Score function to rank the docked ligands and the iterated simplex algorithm as the search method. For SmCB1, the docking grid was defined as a virtual binding sphere with a 15 Å radius, centered at the coordinates X: −20.75, Y: 11.81, and Z: −21.70 Å. The resulting MolDock scores were used as quantitative indicators of binding strength. The top-ranked binding pose for each ligand–protein complex was subsequently examined and visualized using the PyMOL Molecular Graphics System v1.3 (http://www.pymol.org) and Discovery Studio Visualizer v. 2026.

### 2.5. Statistical Analysis

Statistical analysis was conducted using GraphPad Prism v10.0 (GraphPad Software, Irvine, CA, USA). Data normality was tested with the Kolmogorov–Smirnov test. Results are presented as mean ± standard deviation (SD). Comparisons between two groups were analyzed using Student’s *t*-test, while multiple group comparisons used one-way ANOVA. Kaplan–Meier survival curves were analyzed using the Log-Rank test. A *p*-value < 0.05 was considered statistically significant.

## 3. Results

### 3.1. EPP-AF^®^ Exhibits In Vitro Schistosomicidal Activity

**Figure 1:**
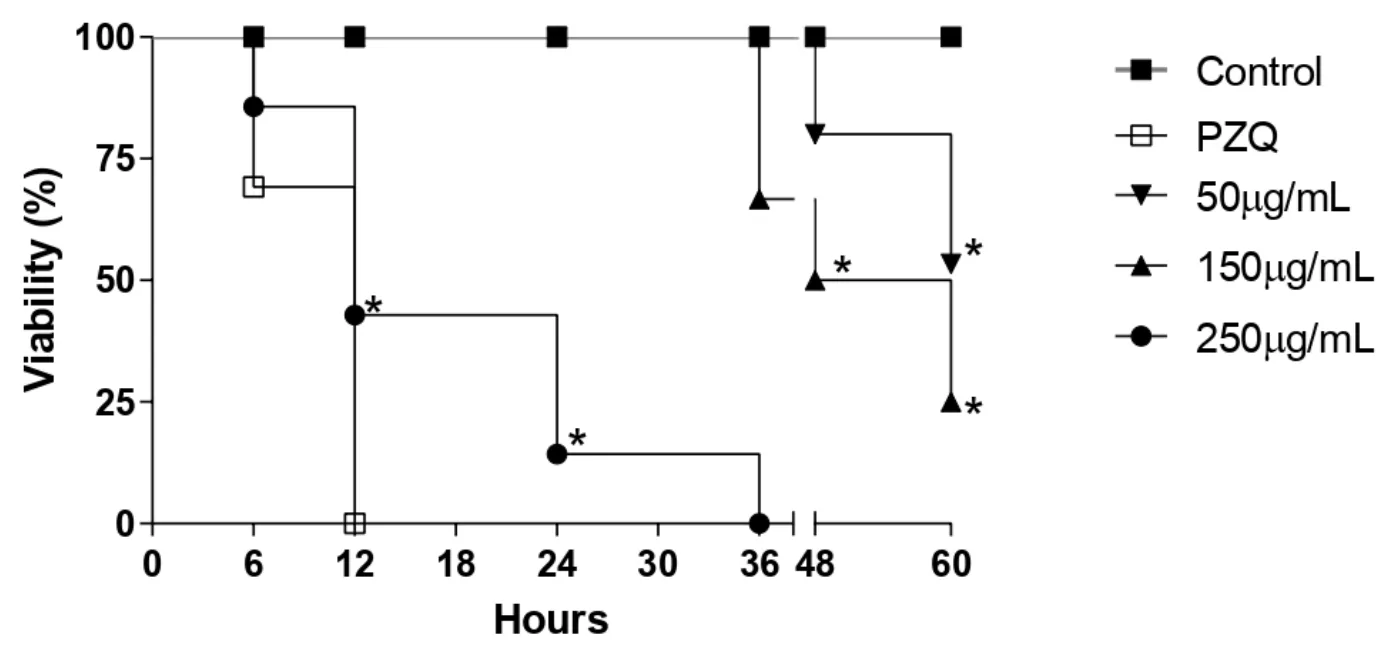
Standardized *A. mellifera* propolis extract (EPP-AF^®^) reduces the viability of adult *S. mansoni* worms. Parasites were exposed to EPP-AF^®^ at concentrations of 50, 150, and 250 μg/mL and compared with the negative control (untreated) and the positive control treated with PZQ (10 μg/mL). Statistical analysis was performed using the Log-Rank test. (*) *p* < 0.05 compared with the negative control.

Exposure of adult *S. mansoni* worms to EPP-AF^®^ caused a concentration- and time-dependent reduction in viability. At 250 µg/mL, viability decreased to 86%, 43%, and 14% after 6, 12, and 24 h, respectively, with complete mortality by 36 h. At 150 µg/mL, viability decreased to 67%, 50%, and 25% at 36, 48, and 60 h, respectively, whereas 50 µg/mL reduced viability to 80% and 53% at 48 and 60 h, without complete elimination. PZQ (10 µg/mL) induced 69% viability at 6 h and complete mortality by 12 h. Sham-treated worms remained fully viable throughout the observation period (Figure 1).

### 3.2. EPP-AF^®^ Reduces Egg Output in Feces and Egg Retention in Liver and Intestine

**Figure 2:**
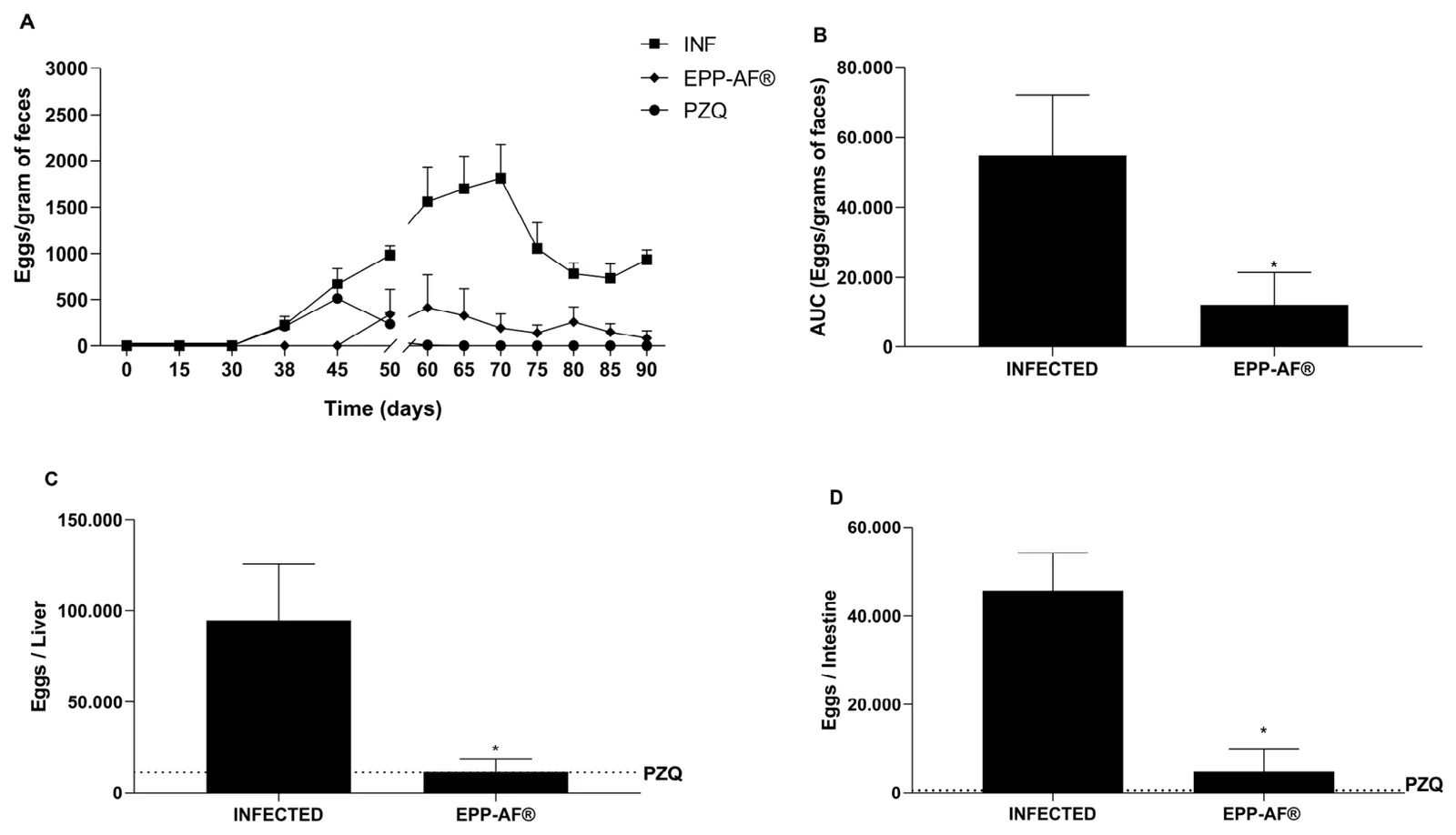
EPP-AF^®^ reduced fecal egg excretion (**A**), egg elimination AUC (**B**), and egg retention in the liver (**C**) and intestine (**D**) of *S. mansoni*-infected animals. Data are mean ± SD (*n* = 6/group). *p* < 0.05 vs. untreated infected control. (*) *p* < 0.05 compared with the negative control.

Administration of EPP-AF^®^ from day 0 to day 30 post-infection delayed and reduced fecal egg shedding (Figure 2A). Eggs were detected from day 50 onward in EPP-AF^®^-treated animals, compared with day 38 in untreated infected controls. AUC analysis confirmed reduced fecal egg excretion between days 60 and 90 (Figure 2B). EPP-AF^®^ also markedly reduced egg retention in the liver, from 94.680 ± 13.778 eggs/g of tissue in infected untreated animals to 11.508 ± 7.291 eggs/g of tissue in EPP-AF^®^-treated animals (Figure 2C). Similarly, intestinal egg retention decreased from 45.700 ± 3.763 eggs/g of tissue in infected untreated animals to 4.942 ± 4.930 eggs/g of tissue following EPP-AF^®^ treatment (Figure 2D).

### 3.3. EPP-AF^®^ Attenuated Tissue Damage and Inflammation in the Liver, Intestine, and Kidneys During S. mansoni Infection

In the liver, EPP-AF^®^ reduced the extent of egg-associated lesions and granulomatous inflammation compared with untreated infected animals. Histopathological evaluation showed lower scores for granuloma presence and extent, fibrosis, tissue damage, necrosis, inflammatory infiltrate, and vascular congestion in EPP-AF^®^-treated animals (Table 1). Similarly, EPP-AF^®^ treatment reduced the scores for the evaluated histopathological parameters in the intestine and kidneys compared with untreated infected animals (Table 1). The histopathological findings were assessed using the predefined ordinal scoring system, ranging from 0 (absent) to 3 (severe), as described in the Methods. Bold formatting removed.

#### 3.3.1. EPP-AF^®^ Reduces Granuloma Counts and Size in Liver, Intestine, and Kidney

**Figure 3:**
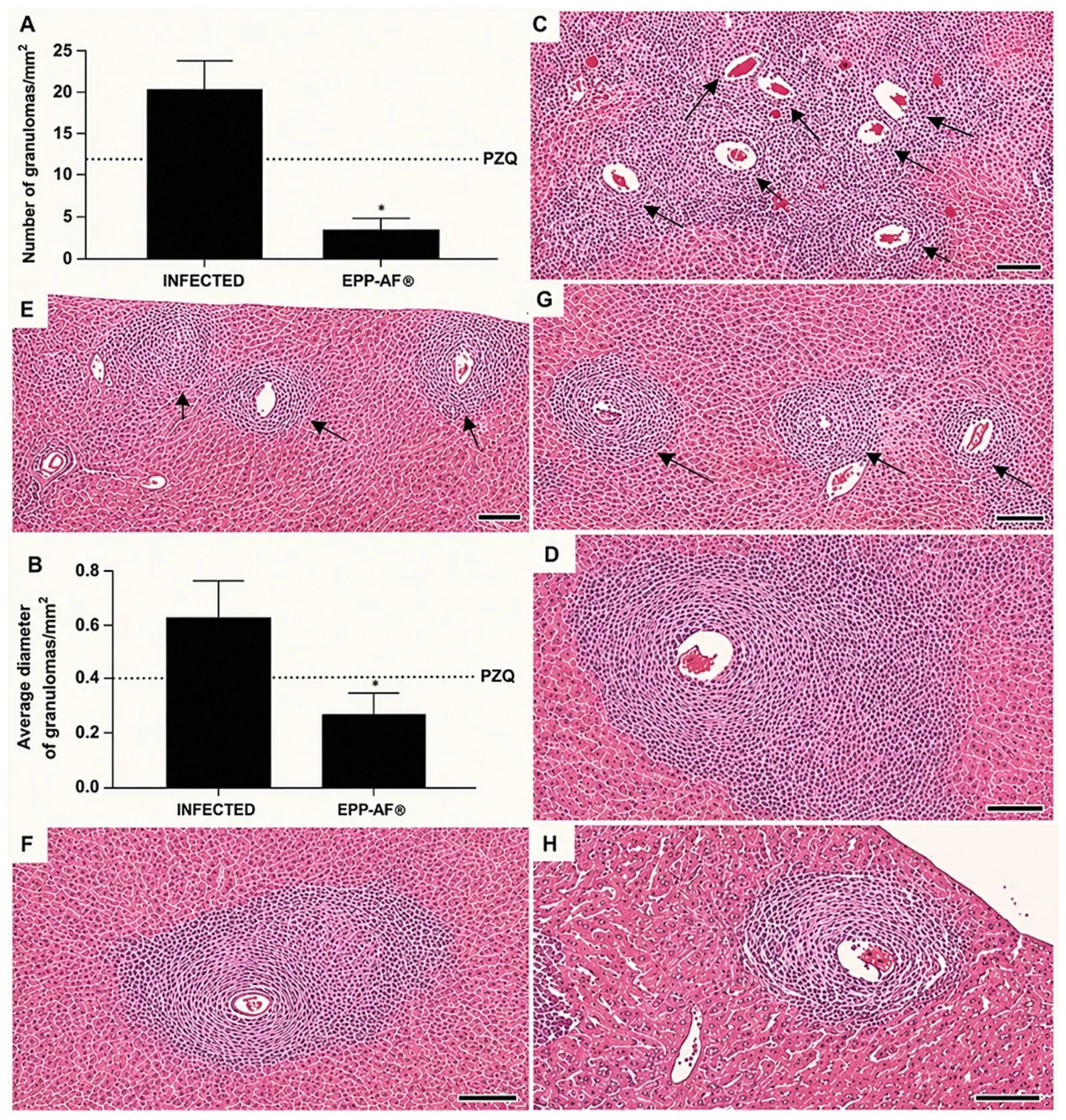
Treatment with EPP-AF^®^ reduced the number (**A**) and diameter (**B**) of hepatic granulomas in mice infected with *S. mansoni* cercariae (70, subcutaneous). Histological sections stained with HE (40×) from Control group: infected and untreated animals (**C**,**D**). PZQ group: Animals treated with Praziquantel^®^ (50 mg/kg) (**E**,**F**) and EPP-AF^®^ group, animals treated with propolis extract for 30 days since day 0 (4.5 mg/Kg, orally) (**G**,**H**). The values represent the mean ± standard deviation of 6 animals per group. (*) *p* < 0.05 compared to the infected control without treatment. The arrows indicate the granulomas in the liver.

**Figure 4:**
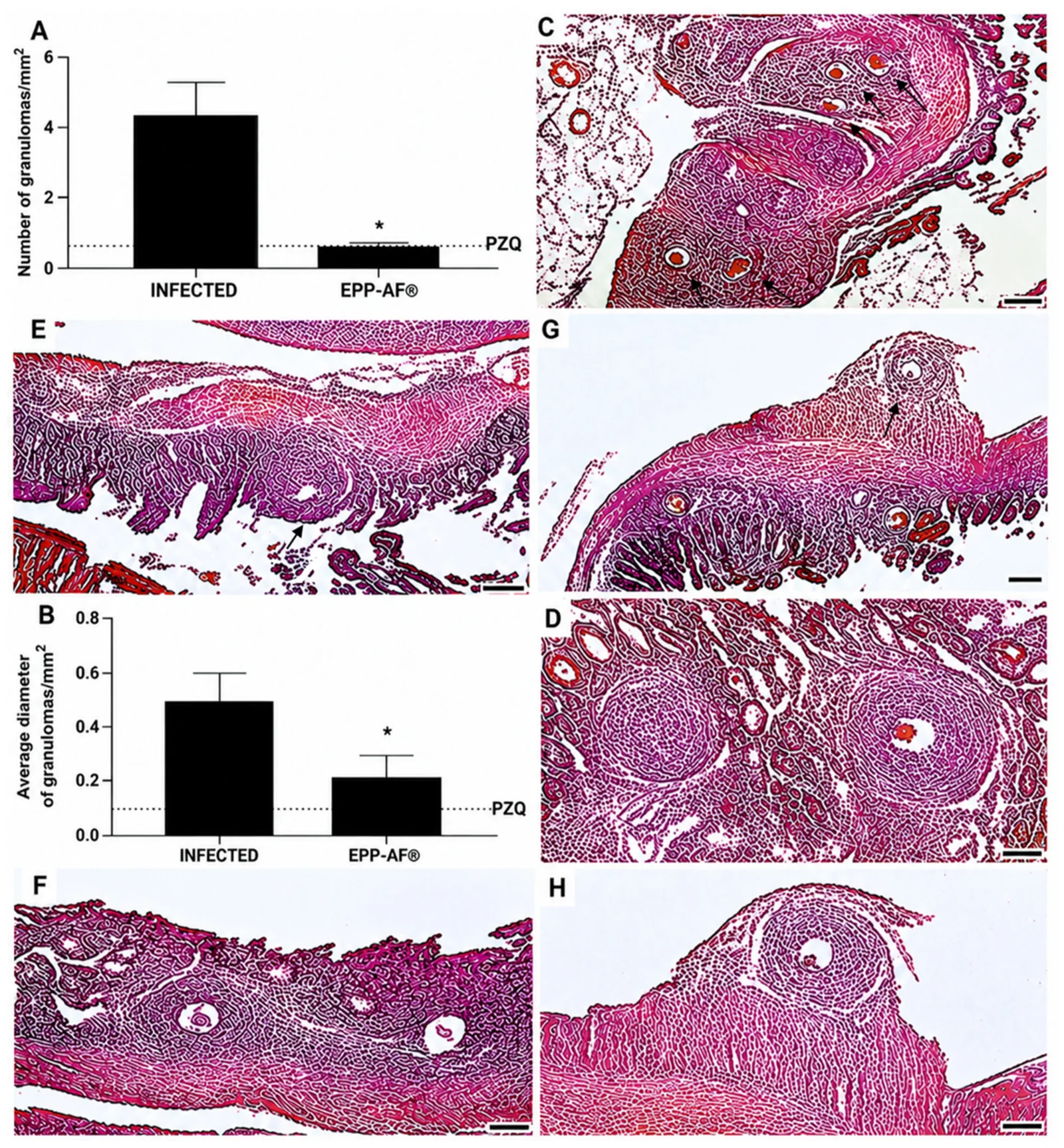
Treatment with EPP-AF^®^ reduced the number (**A**) and diameter (**B**) of intestinal granulomas in mice infected with *S. mansoni* cercariae (70, subcutaneous). Histological sections stained with HE (40×) from Control group: infected and untreated animals (**C**,**D**). PZQ group: Animals treated with Praziquantel^®^ (50 mg/kg) (**E**,**F**) and EPP-AF^®^ group, animals treated with propolis extract for 30 days since day 0 (4.5 mg/Kg, orally) (**G**,**H**). The values represent the mean ± standard deviation of 6 animals per group. (*) *p* < 0.05 compared to the infected control without treatment. The arrows indicate the granulomas in the intestine.

**Figure 5:**
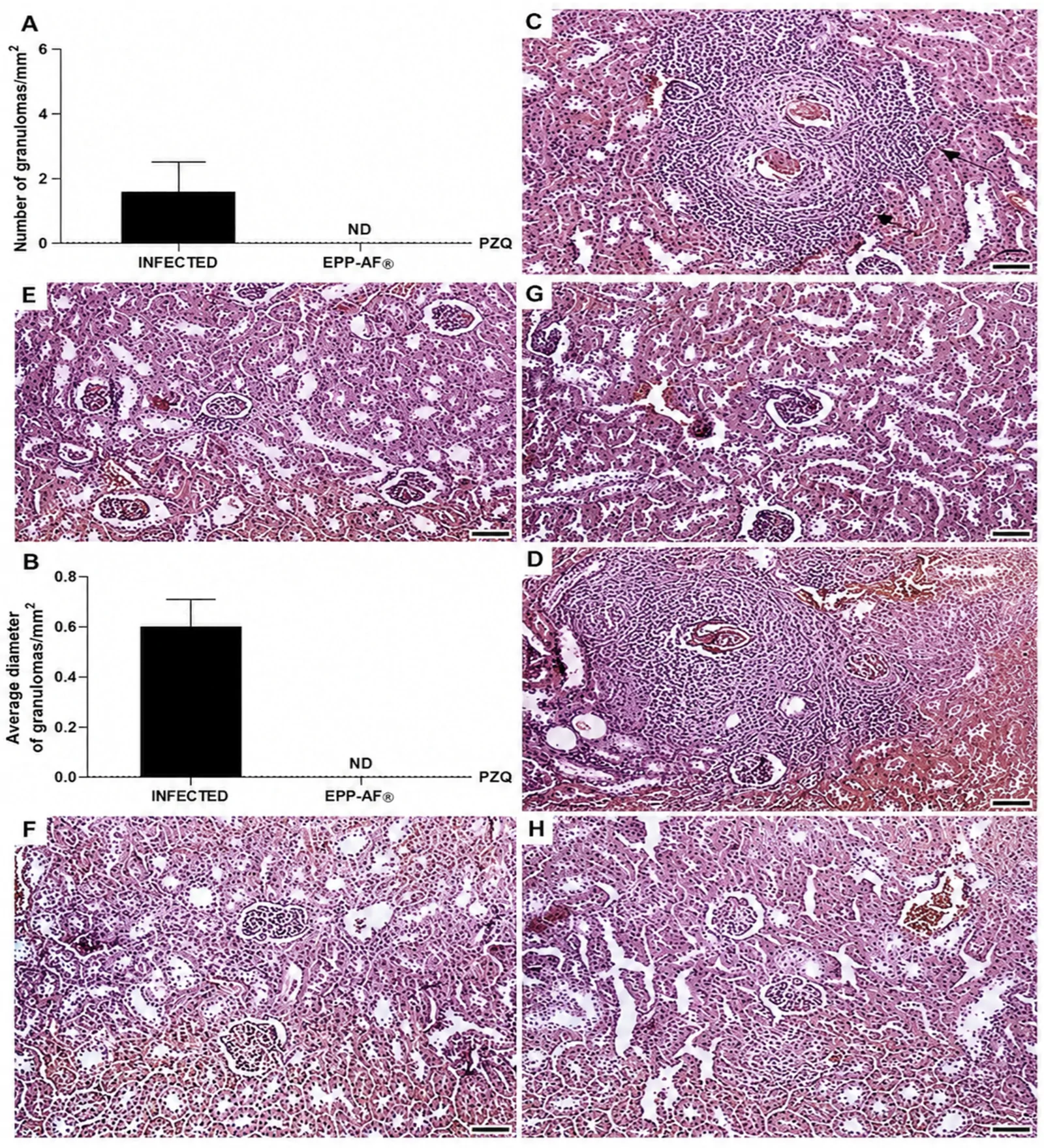
Treatment with EPP-AF^®^ reduced the number (**A**) and diameter (**B**) of renal granulomas in mice infected with *S. mansoni* cercariae (70, subcutaneous). Histological sections stained with HE (40×) from Control group: infected and untreated animals (**C**,**D**). PZQ group: Animals treated with Praziquantel^®^ (50 mg/kg) (**E**,**F**) and EPP-AF^®^ group, animals treated with propolis extract for 30 days since day 0 (4.5 mg/Kg, orally) (**G**,**H**). The values represent the mean ± standard deviation of 6 animals per group. ND—not detected.

Treatment with EPP-AF^®^ reduced both the number and diameter of hepatic (Figure 3), intestinal (Figure 4), and renal (Figure 5) granulomas. Histological sections revealed a lower number of inflammatory cells surrounding the eggs and better-preserved tissue architecture in mice treated with EPP-AF^®^ and PZQ compared to the infected control.

#### 3.3.2. Epp-Af^®^ Modulates Leukocyte Response During Infection

At day 30 post-infection, EPP-AF^®^ reduced eosinophilia. In contrast, the Control animals showed elevated neutrophil, lymphocyte, and eosinophil counts, indicating systemic inflammation. By day 90, both treatments lowered eosinophil and monocyte counts while increasing neutrophils, suggesting immunomodulatory effects even during chronic infection (Table 2).

**Figure 6:**
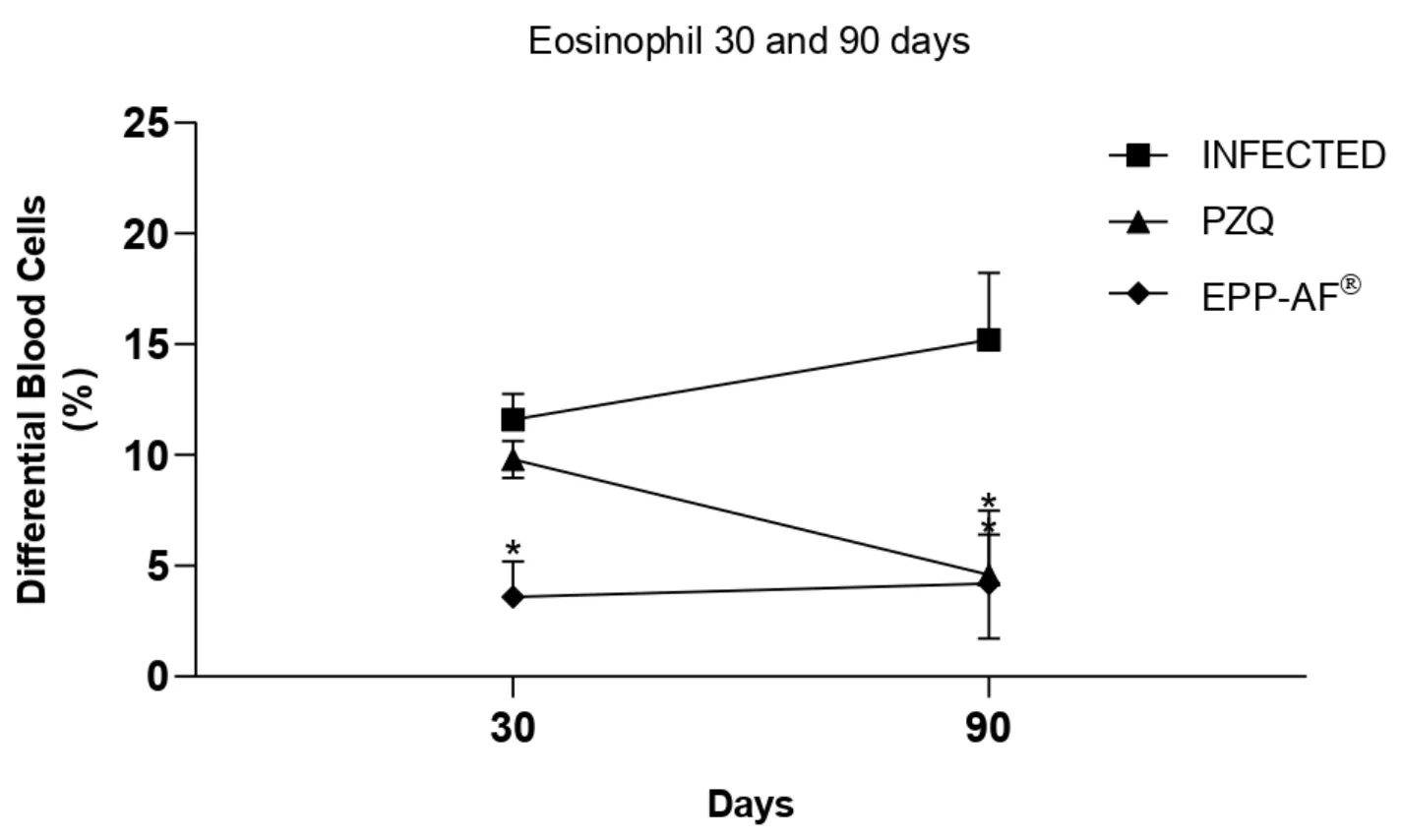
Percentage of eosinophils in peripheral blood at 30 and 90 days after subcutaneous infection with *S. mansoni* cercaria (70). Data are presented as mean ± standard deviation (*n* = 6 per group). (*) *p* < 0.05 vs. untreated infected control.

Figure 6 shows that eosinophilia was significantly lower in the EPP-AF^®^ group from day 30 to day 90 compared to controls. At 90 days, eosinophil levels remained reduced in the EPP-AF^®^ group.

#### 3.3.3. EPP-AF^®^ Reduces Bone Marrow and Peritoneal Cellularity

EPP-AF^®^ reduced cellularity in the bone marrow and peritoneum 90 days post-infection compared to the control group, in which an increase in leukocyte count was observed (Table 3).

#### 3.3.4. Treatment with EPP-AF^®^ Impacts Splenic Cellularity and Cell Population

**Figure 7:**
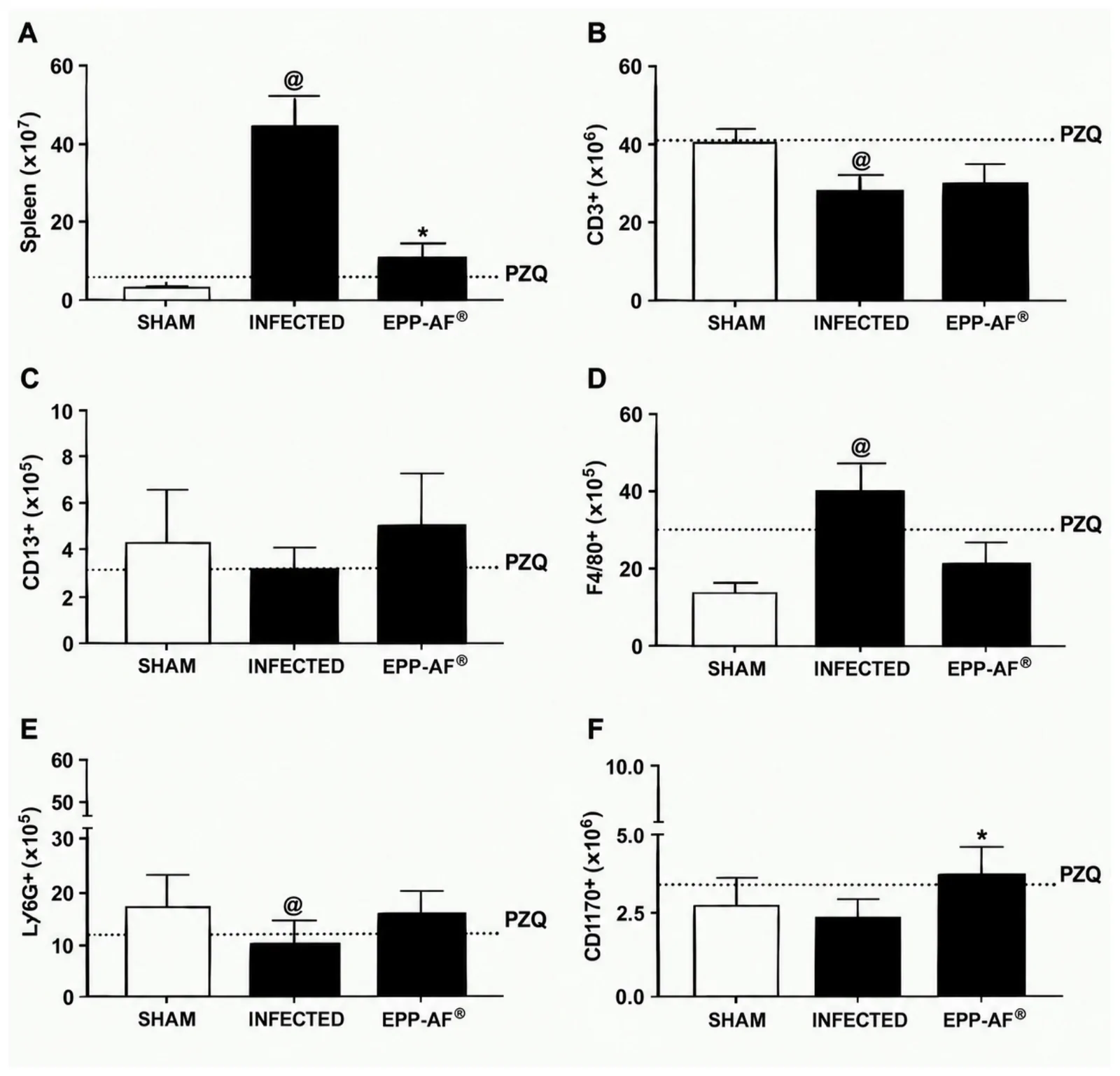
Effect of standardized propolis extract from *A. mellifera* (EPP-AF^®^) on total spleen cells (**A**) and T cells (**B**), B lymphocytes (**C**), macrophages (**D**) neutrophils (**E**) and eosinophils (**F**) distribution. The cells obtained on day 90, belongs from animals infected with *S. mansoni* cercariae (70, subcutaneously). The group EPP-AF^®^ received the extract orally, for 30 days, from day 0 to day 30 post-infection. The group PZQ received the PZQ orally, during seven days, between days 45 and 51 post-infection. The Control group did not receive treatment. The values represent the mean ± standard deviation of six animals per group. (^@^) *p* < 0.05 vs. Sham; (*) *p* < 0.05 compared to the control group.

Total splenic cellularity was reduced in the EPP-AF^®^ group compared to controls (Figure 7A). Flow cytometry revealed a reduction in splenic macrophages (Figure 7D) in both treated groups, with values close to those observed in the *Sham* group. Interestingly, there was an increase in eosinophils in the EPP-AF^®^ group (Figure 7F), suggesting selective recruitment. Other subpopulations (T cells, B cells, and neutrophils) showed no significant differences between the groups (Figure 7B,C,E).

#### 3.3.5. EPP-AF^®^ Modulates Cytokine and Chemokine Production

**Figure 8:**
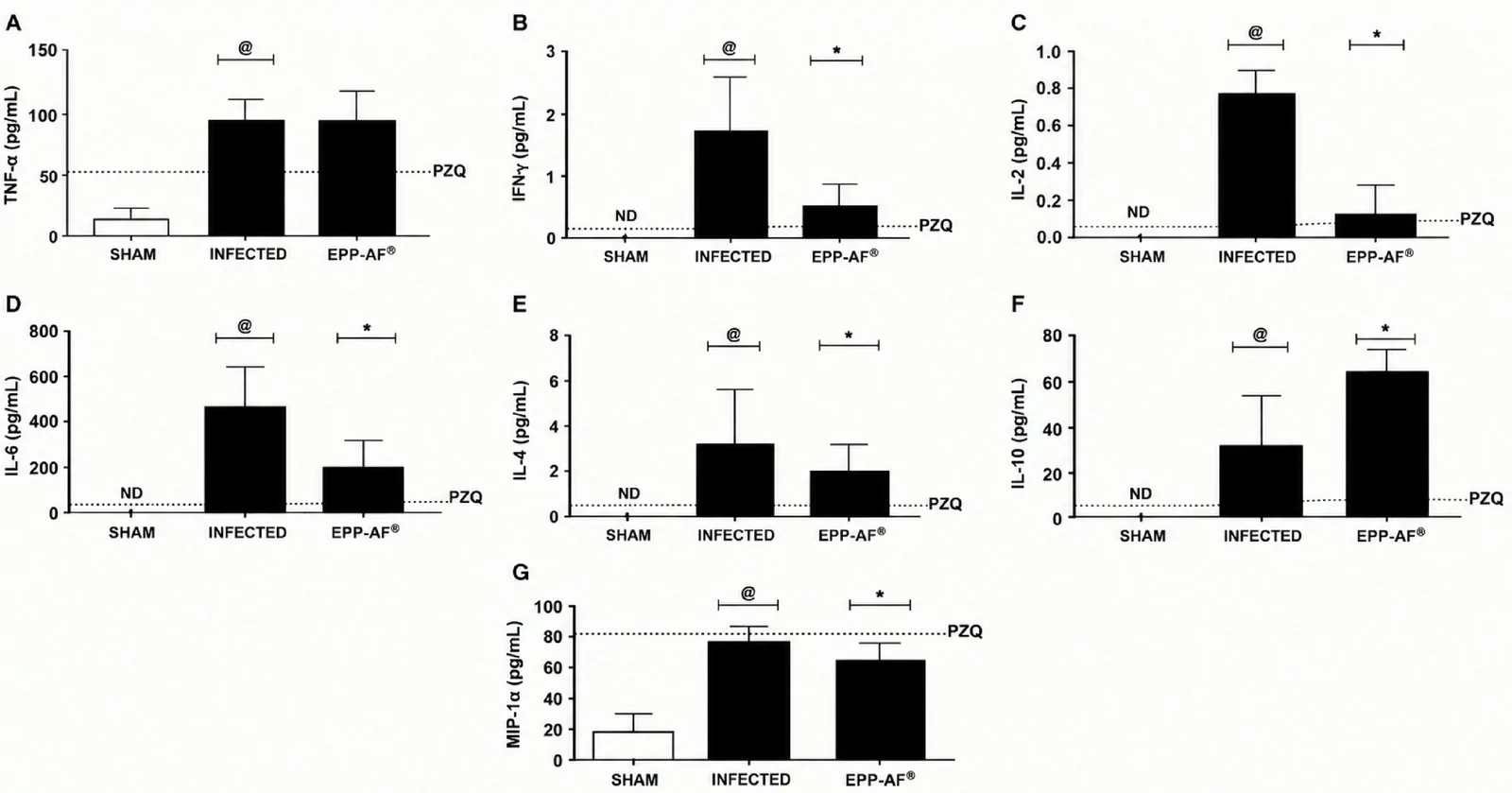
EPP-AF^®^ effect on cytokine levels. TNF-α (**A**) IFN-γ (**B**), IL-2 (**C**), IL-6 (**D**) IL-4 (**E**), IL-10 (**F**) and the chemokine MIP-1 (**G**) present in the supernatant of splenic cell cultures. The cells obtained on day 90, belongs from animals infected with *S. mansoni* cercariae (70, subcutaneously). The group EPP-AF^®^ received the extract orally, for 30 days, from day 0 to day 30 post-infection. The group PZQ received the PZQ orally, during seven days, between days 45 and 51 post-infection. The Control group did not receive treatment. The values represent the mean ± standard deviation of six animals per group. (^@^) *p* < 0.05 vs. Sham; (*) *p* < 0.05 compared to the control group. ND—Not detectable.

EPP-AF^®^ did not alter TNF-α levels (Figure 8A) but reduced levels of IFN-γ (Figure 8B), IL-2 (Figure 8C), and IL-6 (Figure 8D) compared to the infected control. Although IL-4 levels remained unchanged (Figure 8E), IL-10—an important anti-inflammatory cytokine—was significantly elevated in the EPP-AF^®^ group (Figure 8F); furthermore, EPP-AF^®^ also reduced levels of the chemokine MIP-1 (Figure 8G).

#### 3.3.6. Analysis Molecular Docking

**Figure 9:**
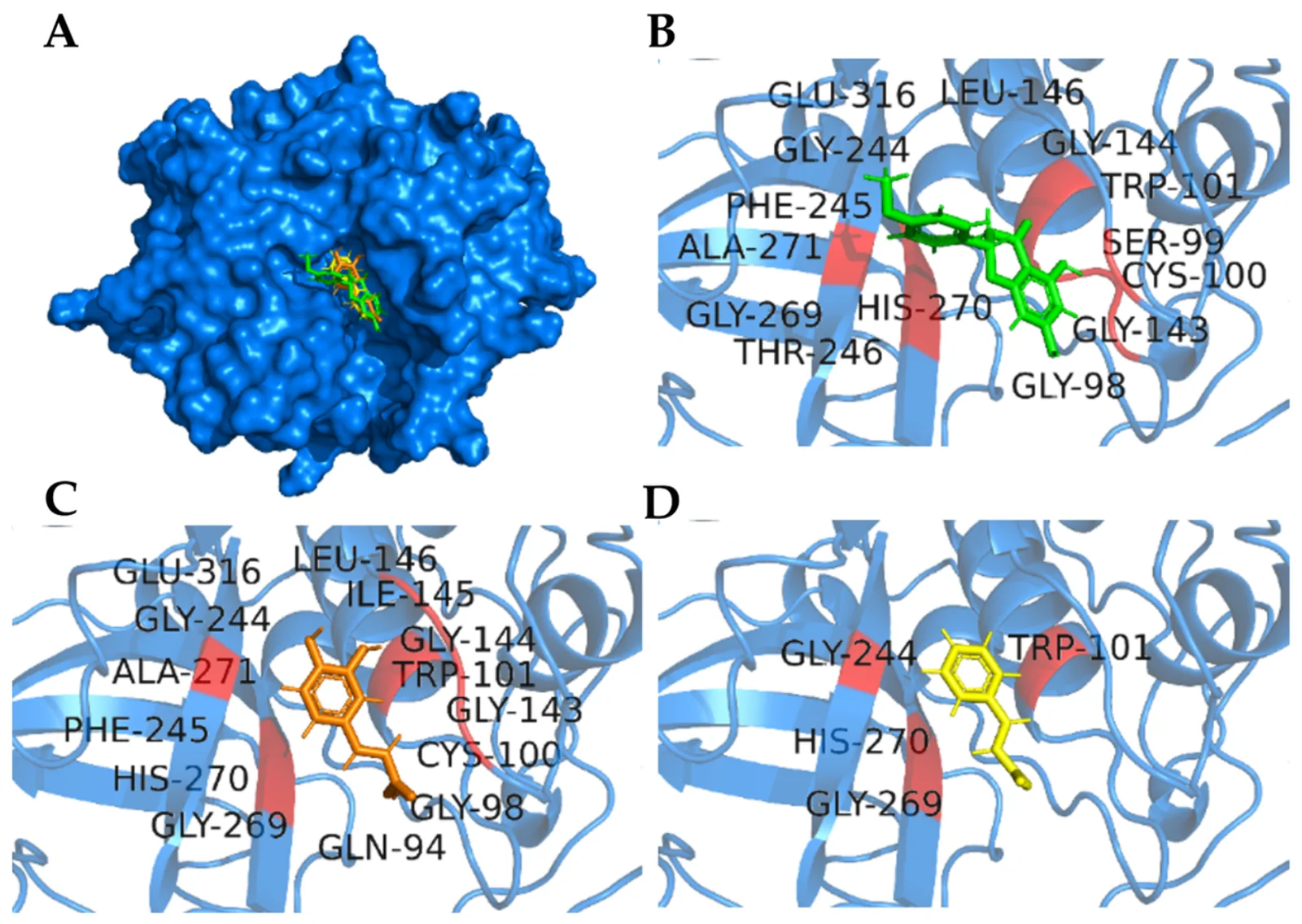
(**A**) Surface representation of Cathepsin B1 from *S. mansoni* (SmCB1), shown in blue. (**B**) Isosakuranetin; (**C**) Caffeic Acid; and (**D**) Cinnamic Acid. The ligands are shown as sticks.

The MolDock scores obtained for the docked compounds were −85.211, −76.369, −70.877, 328.485, 501.969, and 504.617 for isosakuranetin, caffeic acid, cinnamic acid, p-coumaric acid, aromadendrin, and artepillin C, respectively (Table 4). The reference inhibitor azanitrile showed the most favorable MolDock score (−116.67). Protein residues located within 3.5 Å of isosakuranetin, caffeic acid, and cinnamic acid in the SmCB1 active site may be involved in the predicted ligand-binding interactions (Figure 9).

## 4. Discussion

The present study demonstrates that the standardized green propolis extract (EPP-AF^®^) derived from *A. mellifera* effectively attenuates the deleterious effects associated with experimental *S. mansoni* infection in mice. Its antiparasitic activity appears to be associated with reduced adult worm viability, impaired oviposition, and decreased egg retention in host tissues, accompanied by attenuation of inflammatory and immunopathological responses. These findings indicate that EPP-AF^®^ exhibits anti-*S. mansoni* activity and oviposition-inhibitory properties, while also promoting tissue protection by limiting infection-associated pathological damage. In vitro, EPP-AF^®^ reduced the viability of adult *S. mansoni* worms in a concentration- and exposure time-dependent manner, with complete lethality observed at the highest extract concentration evaluated. These findings are consistent with previous studies investigating Brazilian green and red propolis extracts, which demonstrated lethal effects against adult worms and significant reductions in egg production. Such effects have been associated with structural damage to parasite eggs, including tegumental disruption and morphological alterations [27].

In infected mice, EPP-AF^®^ treatment delayed the onset of fecal egg shedding, which was first detected at day 50 post-infection, compared with untreated animals, in which egg elimination occurred earlier (day 30 post-infection). Moreover, treatment consistently reduced egg output throughout the experimental period, extending until day 90. These results suggest that EPP-AF^®^ exerts an early antiparasitic effect, potentially by interfering with parasite development and reproductive processes, consequently reducing egg burden in feces and tissues, including the liver, intestine, and kidneys. These observations agree with previous studies demonstrating reductions in immature egg burdens following exposure to different propolis extracts [25,27,44,45].

Although the reduction in egg burden and tissue pathology indirectly indicates a possible decrease in parasite burden, adult worm numbers were not directly quantified in vivo through perfusion or parasite recovery procedures. This methodological decision was based on the prioritization of comprehensive histopathological and immunological assessments, considering the limited number of experimental animals and ethical constraints associated with animal use.

Egg retention within host tissues represents a major determinant of granuloma formation and progressive organ damage during schistosomiasis. Approximately 40% of parasite-produced eggs remain trapped in host tissues, particularly in the liver and intestine, where they induce predominantly Th2-type immune responses associated with granuloma formation and fibrosis progression [4,46,47]. Morphometric analyses demonstrated that treatment with EPP-AF^®^ reduced the number and size of granulomas in the liver, kidneys, and intestine. Furthermore, histopathological analyses revealed a reduction in scores associated with fibrosis, tissue damage, necrosis, inflammatory infiltration, and vascular congestion. These findings indicate a protective effect of EPP-AF^®^, likely related to the reduction in parasite-induced inflammation and the previously described anti-inflammatory properties of this extract [48,49,50]. Supporting these observations, EPP-AF^®^ administration was associated with increased IL-10 production and modulation of inflammatory cytokine profiles, including reduced levels of IL-6, IFN-γ, and IL-2, in splenic cell cultures. Similar tissue-protective effects have been reported following propolis administration, including reduced collagen accumulation and attenuation of hepatic injury [21,45].

Collectively, these findings suggest that EPP-AF^®^ contributes to the regulation of inflammatory responses during *S. mansoni* infection. However, additional investigations are necessary to determine whether these effects result from direct immunomodulatory mechanisms or occur primarily as a consequence of reduced parasite burden. Beyond its antiparasitic properties, EPP-AF^®^ influenced host immune cell profiles, including alterations in eosinophil, macrophage, and lymphocyte populations in immunologically relevant organs and tissues. These findings are particularly relevant considering that *S. mansoni* infection induces intense inflammatory responses and pronounced Th2 polarization, mainly driven by parasite egg antigens and associated with progressive fibrosis [51,52,53]. Therefore, the immune alterations observed following EPP-AF^®^ administration may reflect both reduced antigenic stimulation due to decreased egg deposition and direct modulation of inflammatory pathways involved in tissue injury.

Eosinophils play an important role in the development and regulation of hepatic Th2 responses [54] and are particularly abundant during the acute phase of *S. mansoni* infection, especially during larval migration and schistosomula development. Although eosinophils exhibit limited activity against adult worms, they display cytotoxic effects against schistosomula through antibody- and complement-dependent mechanisms [55]. The reduction in eosinophil levels observed following EPP-AF^®^ administration suggests attenuation of eosinophil-associated inflammation during infection; however, potential direct effects on early parasite developmental stages require further investigation.

Furthermore, EPP-AF^®^ modified immune cellular profiles in the bone marrow, peritoneal cavity, and spleen, including reduced splenic macrophage populations (F4/80^+^) and increased expression of eosinophil-lineage-associated markers (CD170^+^). This selective modulation may represent an adaptive adjustment of immune homeostasis associated with reduced parasite burden and preservation of tissue integrity. Previous studies have demonstrated that green propolis can influence Th2-associated immune responses and reduce eosinophilic inflammation in murine models of inflammatory pulmonary disorders [49,56]. To our knowledge, this represents the first study describing the effects of EPP-AF^®^ during *S. mansoni* infection through an integrated evaluation of parasitological, histopathological, inflammatory, and immunological parameters.

Additional evidence supporting the association between EPP-AF^®^ administration and inflammatory regulation was obtained through cytokine analyses, which demonstrated modulation of the inflammatory profile, characterized by reduced levels of IFN-γ, IL-2, IL-6, and MIP-1α, together with increased IL-10 concentrations. IL-10 plays a central role in controlling excessive inflammatory responses and limiting tissue damage, acting synergistically with TGF-β to reduce hepatic injury and improve survival during *S. mansoni* infection [57]. Furthermore, IL-4-mediated expansion of IL-10-producing Foxp3^−^ T cells has been shown to regulate egg-induced granulomatous inflammation [58].

MIP-1α is a chemokine involved in the recruitment of inflammatory cells to sites of egg deposition and plays a critical role in granuloma formation [59]. Therefore, its reduction following EPP-AF^®^ treatment may contribute to the decreased granuloma size and number observed in treated animals. Previous studies have demonstrated that elevated plasma MIP-1α levels are associated with severe hepatosplenic schistosomiasis, suggesting that reduced expression of this chemokine may contribute to limiting disease progression [60].

The use of splenic cell supernatants in the present study was based on the fundamental role of the spleen as a secondary lymphoid organ involved in systemic immune regulation during schistosomiasis. The analysis of splenic cell responses provides relevant information regarding immune mechanisms mediated by activated cell populations following infection and represents an established strategy for investigating immunological alterations in murine experimental models. This approach allowed the correlation between cytokine production profiles and alterations in splenic immune cell populations, rather than relying exclusively on serum measurements or tissue homogenates. Liver homogenates were not evaluated due to methodological limitations associated with sample preservation and stability under the experimental conditions, as well as the requirement to preserve hepatic tissue for egg quantification and histopathological analyses.

Molecular docking analyses provided additional evidence that phenolic compounds present in propolis may interact with SmCB1, a cysteine protease of *S. mansoni*. Among the evaluated compounds, isosakuranetin, caffeic acid, and cinnamic acid exhibited the most negative MolDock scores, indicating stronger predicted interactions and suggesting potential inhibitory activity against this enzymatic target. These interactions involved amino acid residues located close to the catalytic region, including GLN94, GLY144, GLY269, GLU316, CYS100, and TRP292, contributing to the stabilization of protein–ligand complexes [61,62]. Nevertheless, molecular docking analyses should be interpreted as predictive evidence of possible ligand–protein interactions rather than direct confirmation of enzymatic inhibition. Further biochemical investigations are necessary to determine whether these compounds effectively inhibit SmCB1 activity and contribute to the antiparasitic effects observed in vivo.

Overall, EPP-AF^®^ demonstrated consistent antiparasitic activity associated with reduced parasite-induced pathology and modulation of inflammatory responses during experimental *S. mansoni* infection. However, the molecular and cellular mechanisms underlying these effects remain incompletely understood. This was expected, considering that the primary objective of this study was to characterize the biological effects of the extract rather than comprehensively elucidate its mechanisms of action. Such investigations would require advanced approaches, including transcriptomic, proteomic, metabolomic, targeted molecular analyses, and the use of genetic models.

Despite these relevant findings, some limitations should be acknowledged. The present study evaluated a single dose of EPP-AF^®^ (4.5 mg/kg) and a fixed treatment duration, representing an initial proof-of-concept investigation. Future dose–response studies are necessary to establish the optimal therapeutic range and determine whether the observed effects occur in a dose-dependent manner. In addition, the follow-up period was limited to 90 days post-infection, a timeframe selected based on previous studies demonstrating its suitability for evaluating the transition from acute to early chronic infection. However, long-term investigations will be required to determine whether the protective effects induced by EPP-AF^®^ persist during advanced stages of chronic schistosomiasis.

Taken together, the findings of the present study provide comprehensive evidence that EPP-AF^®^ exerts multifactorial protective effects during experimental *S. mansoni* infection, combining antiparasitic activity with the regulation of host inflammatory and immune responses. The extract reduced egg-associated tissue damage, attenuated granulomatous inflammation and fibrosis, and promoted a more balanced immune environment, characterized by increased IL-10 production and modulation of inflammatory pathways. Although the precise molecular mechanisms underlying these effects remain to be elucidated, the integration of parasitological, histopathological, immunological, and molecular docking approaches reinforces the potential of EPP-AF^®^ as a bioactive natural product capable of limiting the progression of schistosomiasis-associated pathology. Future studies involving dose–response evaluations, mechanistic investigations, and long-term experimental models will be essential to determine its therapeutic applicability and optimize its use as a complementary strategy for schistosomiasis control.

## 5. Conclusions

In conclusion, this study demonstrates that standardized green propolis extract (EPP-AF^®)^, derived from *A. mellifera*, exhibits significant antiparasitic, anti-inflammatory, and immunomodulatory effects during experimental *S. mansoni* infection in mice. EPP-AF^®^ reduced adult worm viability, delayed and decreased egg elimination, limited tissue damage induced by egg deposition, and modulated immune responses associated with inflammation and fibrosis. These findings highlight the potential of EPP-AF^®^ as a promising natural-derived candidate for reducing schistosomiasis-associated pathology and support further investigations aimed at elucidating its mechanisms of action and evaluating its potential therapeutic application.

## Figures and Tables

**Table 1:** Histological analysis of liver, intestine and kidney from animals infected with *S. mansoni* cercariae, treated with propolis (EPP-AF^®^).

	Liver				Intestine				Kidney			
Groups	Sham	Infected	PZQ	EPP-AF^®^	Sham	Infected	PZQ	EPP-AF^®^	Sham	Infected	PZQ	EPP-AF^®^
No. eggs	0.0 ± 0.0	3.0 ± 0.0 ^@^	2.0 ± 0.0 *	1.3 ± 1.5 *	0.0 ± 0.0	3.0 ± 0.0 ^@^	0.3 ± 0.5 *	0.6 ± 1.0 *	0.0 ± 0.0	1.3 ± 1.0 ^@^	0.0 ± 0.0 *	0.0 ± 0.0 *
No. Granulomas	0.0 ± 0.0	3.0 ± 0.0 ^@^	2.0 ± 0.0 *	2.0 ± 1.7	0.0 ± 0.0	3.0 ± 0.0 ^@^	0.3 ± 0.5 *	0.6 ± 1.0 *	0.0 ± 0.0	1.3 ± 1.0 ^@^	0.0 ± 0.0 *	0.0 ± 0.0 *
Granuloma area	0.0 ± 0.0	3.0 ± 0.0 ^@^	2.0 ± 0.0 *	1.3 ± 1.5 *	0.0 ± 0.0	1.6 ± 0.5 ^@^	0.3 ± 0.5 *	0.3 ± 0.5 *	0.0 ± 0.0	1.1 ± 0.09 ^@^	0.0 ± 0.0 *	0.0 ± 0.0 *
Fibrosis	0.0 ± 0.0	2.0 ± 0.0 ^@^	1.0 ± 0.0 *	0.6 ± 0.5 *	0.0 ± 0.0	1.3 ± 0.5 ^@^	0.3 ± 0.5 *	0.3 ± 0.5 *	0.0 ± 0.0	0.6 ± 1.0 *	0.0 ± 0.0 *	0.0 ± 0.0 *
Tissue damage	0.0 ± 0.0	3.0 ± 0.0 ^@^	2.0 ± 0.0 *	1.0 ± 0 *	0.0 ± 0.0	3.0 ± 0.0 ^@^	1.3 ± 0.5 *	1.3 ± 0.5 *	0.0 ± 0.0	1.6 ± 0.5 ^@^	1.0 ± 0.0 *	0.0 ± 0.0 *
Necrosis	0.0 ± 0.0	1.3 ± 0.5 ^@^	1.0 ± 0.0 *	1.0 ± 0	0.0 ± 0.0	1.3 ± 0.5 ^@^	0.0 ± 0.0 *	0 ± 0.0 *	0.0 ± 0.0	0.3 ± 0.5 ^@^	0.0 ± 0.0 *	0.0 ± 0.0 *
Cell infiltration	0.0 ± 0.0	3.0 ± 0.0 ^@^	1.6 ± 0.5 *	2.0 ± 0 *	0.0 ± 0.0	3.0 ± 0.0 ^@^	1.6 ± 0.5 *	2 ± 0.0 *	0.0 ± 0.0	1.6 ± 0.5 ^@^	1.0 ± 0.0 *	0.6 ± 1.0 *
Vascular congestion	0.0 ± 0.0	2.0 ± 0.0 ^@^	1.3 ± 0.5 *	0.6 ± 0.5 *	0.0 ± 0.0	3.0 ± 0.0 ^@^	0.6 ± 0.5 *	0.3 ± 0.5 *	0.0 ± 0.0	0.0 ± 0.0	0.0 ± 0.0	0.0 ± 0.0

The animals were infected with *S. mansoni* with 70 cercariae via subcutaneous inoculation and treated with EPP-AF^®^ (4.5 mg/kg, oral) for 30 consecutive days. The PZQ group was treated with Praziquantel^®^ (PZQ) (50 mg/kg, oral) for 7 days, between the 45th and 51st day after infection. The animals in the control group included infected and untreated animals, and the Sham group included uninfected and untreated animals. Values represent mean ± SD (*n* = 6/group). (^@^) *p* < 0.05 vs. Sham; (*) *p* < 0.05 vs. untreated infected control.

**Table 2:** Differential leukocyte counts (%) in the blood of mice at 30 and 90 days after infection with *S. mansoni*.

Time	30 Days—Differential Leukocyte Count (%)				90 Days—Differential Leukocyte Count (%)			
Group/Cells	Sham	Infected	PZQ	EPP-AF^®^	Sham	Infected	PZQ	EPP-AF^®^
Leukocytes	100	100	100	100	100	100	100	100
Neutrophils	26.8 ± 3.4	7.6 ± 5.1 ^@^	11.0 ± 1.8 ^@^	12.2 ± 7.4 ^@^	30.6 ± 5.4	14.4 ± 2.9 ^@^	12.4 ± 6.0	21.0 ± 12.8
Lymphocytes	60.0 ± 5.6	74.2 ± 3.9 ^@^	68.0 ± 3.9	54.2 ± 31.9	58.2 ± 2.5	60.6 ± 4.2	75.2 ± 7.4 *	68.2 ± 13.7
Monocytes	11 ± 1.2	6.6 ± 2.7	7.2 ± 1.4	5.6 ± 5.0	11.8 ± 0.8	10.2 ± 1.6	6.0 ± 3.0 ^@^	2.2 ± 1.6 *^@^
Eosinophils	1.4 ± 1.1	11.6 ± 2.2 ^@^	9.8 ± 0.8 ^@^	3.6 ± 3.5 *	0.2 ± 0.4	15.2 ± 6.7 ^@^	4.6 ± 2.8 *^@^	4.2 ± 4.9 *^@^

The animals were infected with *S. mansoni* with 70 cercariae via subcutaneous inoculation and treated with EPP-AF^®^ (4.5 mg/kg, oral) for 30 consecutive days. The PZQ group was treated with Praziquantel^®^ (PZQ) (50 mg/kg, oral) for 7 days, between the 45th and 51st day after infection. The animals in the control group included infected and untreated animals, and the Sham group included uninfected and untreated animals. Values represent mean ± SD (*n* = 6/group). (^@^) *p* < 0.05 vs. Sham; (*) *p* < 0.05 vs. untreated infected control.

**Table 3:** Leukocyte count of bone marrow and peritoneum of mice infected with *S. mansoni* and treated with propolis (EPP-AF^®^).

Groups Cells (×10^6^/mL)	Sham	Infected	PZQ	EPP-AF^®^
Bone marrow	1.1 ± 0.2	15.2 ± 8.0 ^@^	0.7 ± 0.6 *	6.1 ± 5.8 *^@^
Peritoneum	2.8 ± 0.2	20.3 ± 4.6 ^@^	5.0 ± 0.5 *^@^	8.11 ± 5.2 *^@^

*S. mansoni*-infected animals received 70 cercariae by subcutaneous inoculation and were treated with EPP-AF^®^ (4.5 mg/kg, oral) for 30 days or PZQ (50 mg/kg, oral) from days 45–51 post-infection. Control animals were infected and untreated, whereas Sham animals were uninfected and untreated. Values are mean ± SD (*n* = 6/group). (^@^) *p* < 0.05 vs. Sham; (*) *p* < 0.05 vs. untreated infected control.

**Table 4:** Virtual screening results for the top-ranked compounds based on MolDock scores against Cathepsin B1 protein from *S mansoni* (SmCB1).

Compound	MolDock Score	Structure
Azanitrile	−116.67	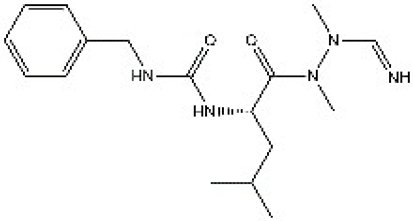
Isosakuranetin	−85.211	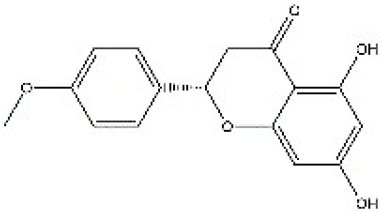
Caffeic Acid	−76.369	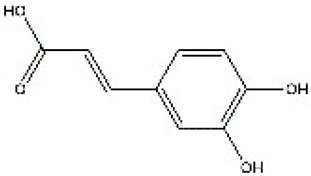
Cinnamic Acid	−70.877	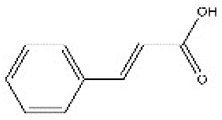
p-Coumaric acid	328.485	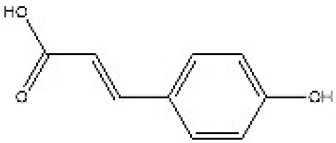
Aromadendrin	501.969	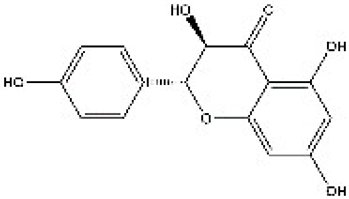
Artepillin C	504.617	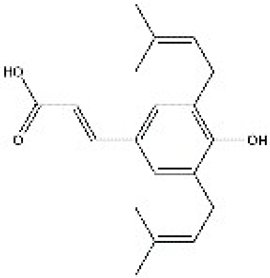

## Data Availability

The original data can be provided upon reasonable request and should be directed to the corresponding author.
